# The diagnostic value of 99m-Tc GSA scintigraphy for liver function and remnant liver volume in hepatic surgery: a retrospective observational cohort study in 27 patients

**DOI:** 10.1186/s13037-018-0161-5

**Published:** 2018-06-04

**Authors:** Naokazu Chiba, Kei Yokozuka, Shigeto Ochiai, Takahiro Gunji, Masaaki Okihara, Toru Sano, Koichi Tomita, Rina Tsutsui, Shigeyuki Kawachi

**Affiliations:** Department of Digestive and Transplantation Surgery, Tokyo Medical University Hachioji Medical Center, 1163 Tatemachi, Hachioji, Tokyo 193-0998 Japan

**Keywords:** Liver kinetic growth, 99mTc-galactosyl human serum albumin scintigraphy, Remnant liver function

## Abstract

**Background:**

The aim was to analyze hepatic hypertrophy after portal vein embolization (PVE) and Associating Liver Partition with Portal vein ligation for Staged hepatectomy (ALPPS) to determine whether clinical circumstances associated with major hepatic resections correlated with remnant growth.

**Methods:**

Data was abstracted from a retrospectively maintained database on 27 patients undergoing hepatic resection followed by PVE and the ALPPS procedure between October 1, 2007 and December 31, 2016. The increasing rate of liver volume and remnant liver LU15 was defined as the percentage-point difference between the liver volume and remnant liver LU15 before and after the intervention or surgery. And correlation between kinetic growth rate (KGR) of liver and future remnant liver volume or remnant liver LU15 was analyzed.

**Results:**

The degree of hypertrophy (DH) of volume and LU15 was significantly greater after ALPPS (volume: 40.3% and LU15: 65.0%) than after PVE (volume: 22.7% and LU15: 48.8%) (*P* < 0.05). KGR of volume and LU15 was significantly greater after ALPPS (volume: 19.0 cm^3^/day and 2.00%/day) (LU15: 0.61 /day and 1.82%/day) than after PVE (volume: 3.89 cm^3^/day and 0.42%/day) (LU15: 0.19 /day and 0.63%/day) (*P* < 0.001). An inverse correlation between KGR and initial remnant liver volume was observed. And a positive correlation between KGR and LU15 was observed.

**Conclusion:**

Future remnant liver volume and KGR was greater after the ALPPS procedure than after PVE. Liver hypertrophy is related to the expected remnant liver volume and total liver function. This study suggested that total liver function and initial remnant liver volume might be a new indication of hepatectomy after PVE and ALPPS in the case of insufficient remnant liver volume.

## Background

Hepatic resection is a potentially curative treatment for a variety of primary hepatobiliary malignancies as well as for numerous metastatic malignancies. As more extensive hepatic resections are performed, achieving adequate remnant liver function often remains the rate limiting step [1]. Assessment of hepatic functional reserve is one of the most important steps in hepatic resection [2].

To estimate hepatic functional reserve, 99mTc-labelled diethylene triamine penta-acetate–galactosyl human serum albumin (99mTc-GSA), a radiopharmaceutical that binds specifically to the hepatic asialoglycoprotein receptor (ASGP-R), has been developed and used clinically to estimate hepatic function [3]. Because ASGP-R is a natural superficial antigen of viable hepatocytes, the uptake of 99mTc-GSA is independent of biochemical processes and allows direct estimation of the functioning hepatocyte mass [4]. In addition, the distribution of 99mTc-GSA in the liver is not dependent on liver blood flow [5]. Our previous study has shown that a novel index, remnant liver LU15, has been identified as a surrogate marker for remnant liver function and preoperative remnant liver LU15 values might predict hepatic failure following a liver resection without biliary reconstruction [6].

Portal vein embolization (PVE) has been repeatedly shown as a reliable technique to induce atrophy of the embolized lobe and compensatory hypertrophy of the future remnant liver, and it currently remains the standard for achieving an appropriate future remnant liver before hepatic resection [7]. The kinetic growth rate (KGR) of future remnant liver after PVE has been reported to be 2.4% per week on average, achieving an increase in future remnant liver from 10 to 46% from 2 to 8 weeks post PVE, respectively [8].

Recently a novel operative approach, the ALPPS procedure (Associating Liver Partition with Portal vein ligation for Staged hepatectomy), has been used to induce hypertrophy of the future liver remnant (FLR) and expedite stages of hepatic resections.

In fact, data show that the standardized future remnant liver has grown by 40–160% in only 6–9 days after ALPPS [9–11]. The mechanisms of the apparent profound hepatic growth of future remnant liver after ALPPS are unknown.

To date, no study has compared the degree and rate of growth of future remnant liver in patients after PVE and ALPPS using a surrogate parameter, remnant liver LU15, by using fusions obtained from contrast-enhanced computed tomography (CT) and 99mTc-GSA single photon emission CT (SPECT). The aim of the present study was to compare the degree and rate of hepatic hypertrophy after PVE and ALPPS to determine whether clinical circumstances associated with major hepatic resections correlated with remnant growth.

## Methods

The retrospective data collection in this study was performed with approval of the Tokyo Medical University Hachioji Medical Center Ethics Committee. Data were abstracted from a retrospectively maintained database on all 27 patients undergoing hepatic resection followed by PVE and the ALPPS procedure between October 1, 2007 and Dec 31, 2016. The indication of hepatectomy for liver metastasis was good function of future remnant liver and without extrahepatic lesions. And the PVE indication was less than 13.0 of remnant liver LU calculated by 99 m-Tc-GSA liver scintigrahy [6]. ALLPS procedure was performed with only the patients, who has insufficient future remnant liver volume and whom was planned surgical procedure of tri-sectionectomy and partial hepatectomy.

All patients with available volumetric data and parameters from 99mTc-GSA liver scintigraphy were included. As part of our standard protocols, patients underwent a CT scan, along with hepatic volumetry and 99mTc-GSA liver scintigraphy with several parameters including remnant liver LU15, before the ALPPS procedure, and 4 days and 7 days after the ALPPS procedure. Total liver LU15 was calculated from the cumulative liver uptake of the tracer 15 to 16 min after an injection of a radiotracer. Remnant liver LU15 was calculated as an index or residual liver function by applying the following equation; Remnant liver LU15 = Total LU15 x residual count ratio (calculated by fusion image of the SPECT images by scintigraphy and a three-dimensional (3D) image of the liver constructed by Synapse Vinscent (Fuji Film, Tokyo, JAPAN)). CT volumetry was performed in patients undergoing PVE immediately prior to embolization and at 3–4 weeks after a PVE just prior to a major hepatic resection. PVE with resected planning branches was used.

Liver volumes were determined by loading CT images onto a Synapse Vincent. The increasing rate of liver volume and remnant liver LU15 was defined as the percentage-point difference between the liver volume and remnant liver LU15 before and after the intervention or surgery (PVE or ALPPS). KGR was calculated as percentage growth per day. [12]

In patients who underwent liver resection, post-operative liver failure was determined according to the International Study Group of Liver Surgery (ISGLS) classification [13]. In this study, grade B and C was defined as postoperative liver failure.Primary end point in this study was the degree of hypertrophy of liver volume and LU15 and correlation of KGR and liver volume or remnant liver LU15.

The degree of hypertrophy of volume and LU15 was significantly greater after ALPPS than after PVE. KGR of volume and LU15 was significantly greater after ALPPS than after PVE. An inverse correlation between KGR and initial remnant liver volume was suggested. And a positive correlation between KGR and LU15 was observed.

All statistical analyses were performed using SPSS package (SPSS 18.0). Differences between groups were analyzed using the unpaired t-test for continuous variables and by the χ2-test or continuity correction method for categorical variables. The Wilcoxon’s rank-sum test was used for variables that did not display a normal distribution. All statistical tests were two-sided, and differences were considered significant when *P* < 0.05.

## Results

We identified 27 patients who underwent PVE and major hepatic resection or the first part of a staged resection and 2 patients who underwent ALPPS procedures. Patient demographics for the two groups are shown in Table 1. The mean age in the PVE group was 69 years and in the ALPPS group was 68 years. Colorectal liver metastasis (CRLM) was the dominant diagnoses for ALPPS group patients, and none of the patients in the ALPPS group had underlying cirrhosis. The PVE group included cholangiocarcionoma (*n* = 10), hepatocellular carcinoma (HCC; *n* = 9), CRLM (*n* = 5) and other disease (*n* = 3). The surgical procedures in the PVE group were hemi-hepatectomy (*n* = 22) and tri-sectionectomy (*n* = 4), which include with pylorus-preserving pancreaticoduodenectomy (PPPD; *n* = 2), bile-duct reconstruction (*n* = 5) and vascular reconstruction (*n* = 5).

**Table 1:** Patients demographics

	PVE (*n* = 27)	ALPPS (*n* = 2)
Age (years)	Median 69	Median 68
Gender (male)	19 (70%)	0 (0%)
Diagnosis		
CRLM	5 (19%)	2 (100%)
Cholangiocarcinoma	10 (37%)	0 (0%)
HCC	9 (33%)	0 (0%)
Others	3 (11%)	0 (0%)
Surgical procedure		
Hr1	1 (4%)	0 (0%)
Hr2	22 (81%)	0 (0%)
Hr3	4 (15%)	0 (0%)
ALPPS	0	2 (100%)
with PPPD	2 (7%)	0 (0%)
with bile-duct reconstruction	10 (37%)	0 (0%)
with vascular reconstruction	5 (19%)	0 (0%)
Liver failure	2 (7%)	0 (0%)
90 day mortality	0 (0%)	0 (0%)

*CRLM* Colorectal liver metastasis, *HCC* Hepatocellular carcinoma, *PPPD* Pylorus-preserving pancreaticoduodenectomy

**Fig. 1:**
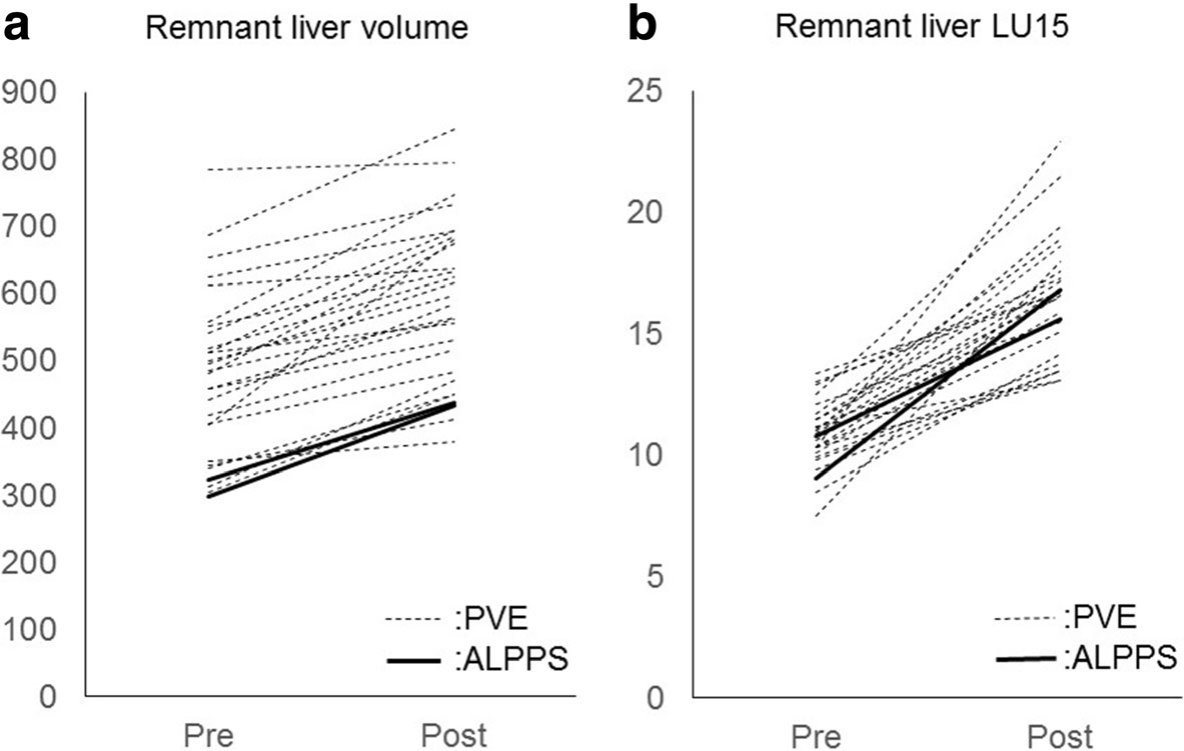
Transition of kinetic liver growth factors determined by volumetry and scintigraphy prior to and after intervention or surgery in two groups. The degree of hypertrophy (DH) of volume and LU15 was significantly greater after ALPPS (volume: 40.3% and LU15: 65.0%) than after PVE (volume: 22.7% and LU15: 48.8%) (*P* < 0.05)

Table 2 summarizes volumetric measurements and LU15 values by 99mTc-GSA liver scintigraphy of remnant liver volume before and after PVE and ALLPPS. Growth of the remnant liver volume and remnant liver LU15 are shown in Fig. 1. The degree of hypertrophy (DH) of volume and LU15 was significantly greater after ALPPS (volume: 40.3% and LU15: 65.0%) than after PVE (volume: 22.7% and LU15: 48.8%) (*P* < 0.05). KGR of volume and LU15 was significantly greater after ALPPS (volume: 19.0 cm^3^/day and 2.00%/day) (LU15: 0.61 /day and 1.82%/day) than after PVE (volume: 3.89 cm^3^/day and 0.42%/day) (LU15: 0.19 /day and 0.63%/day) (*P* < 0.001).

**Table 2:** Future remnant liver growth

	PVE (*n* = 27)	ALPPS (*n* = 2)
Total liver volume (post - pre) (cc)	− 73	− 91
Remnant liver volume (post - pre) (cc)	100	124
LHL15 (post - pre)	0.0055	−0.001
HH15 (post - pre)	0.006	0
LU15 (post - pre)	0.8	−0.94
Remnant liver LU15 (post - pre)	5.75	6.28
Time interval (days)	31	10
Remnant liver volume DH (%)	22.7	40.3
Remnant liver LU15 DH (%)	48.8	65.0
KGR (cc/day)	3.89	19.0
KGR (%/day)	0.42	2.00
Remnant liver LU15 KGR (/day)	0.19	0.61
Remnant liver LU15 KGR (%/day)	0.63	1.82

All values are median; *DH* degree hypertrophy, *KGR* kinetic liver growth rate

**Fig. 2:**
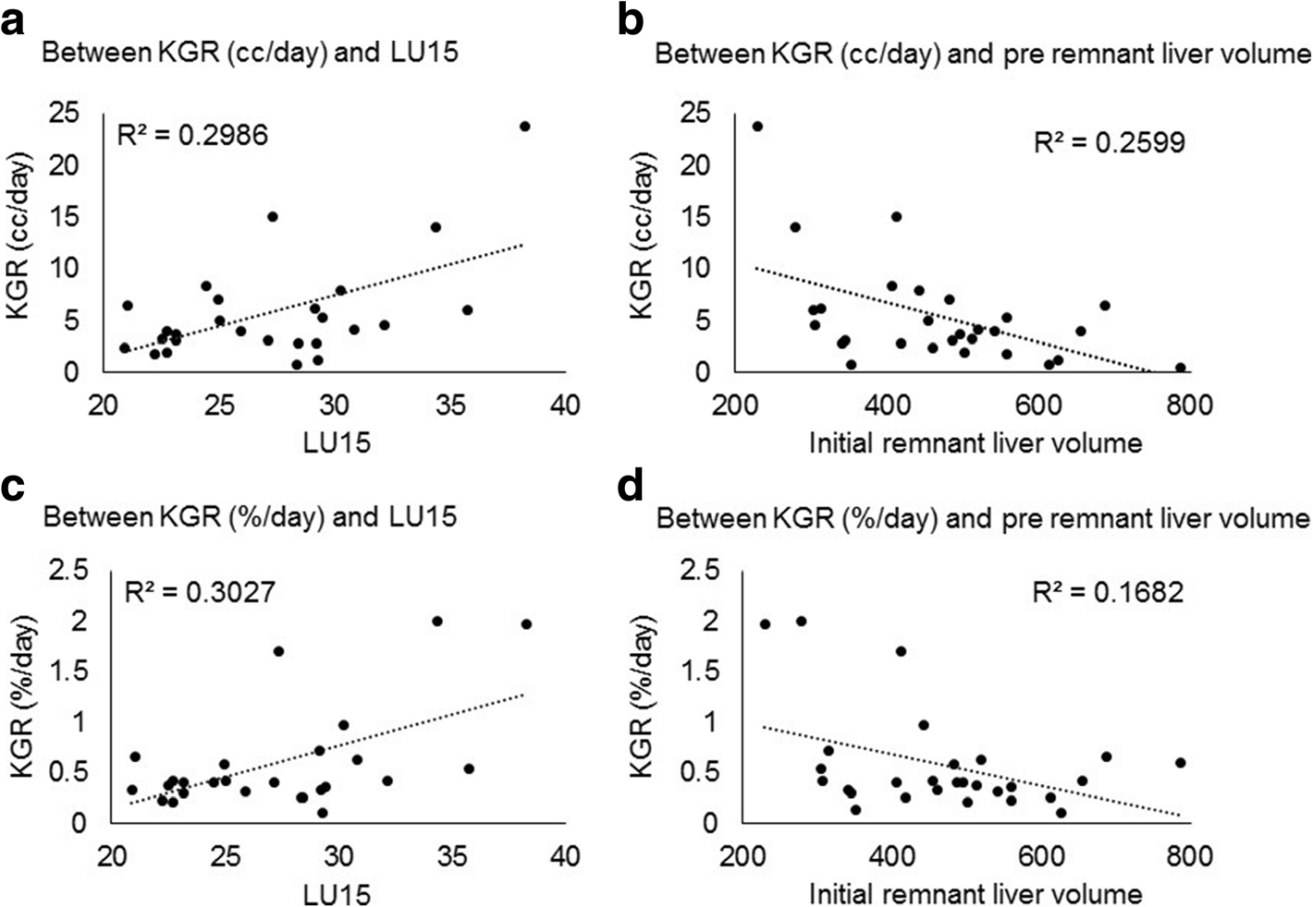
Correlation between kinetic liver growth rate (KGR) of liver volume and LU15 or initial remnant liver volume. An inverse correlation between KGR of liver volume and initial remnant liver volume was suggested. KGR of liver volume decreased with increasing of the initial remnant liver volume. A positive correlation between KGR of liver volume and LU15 was observed

**Fig. 3:**
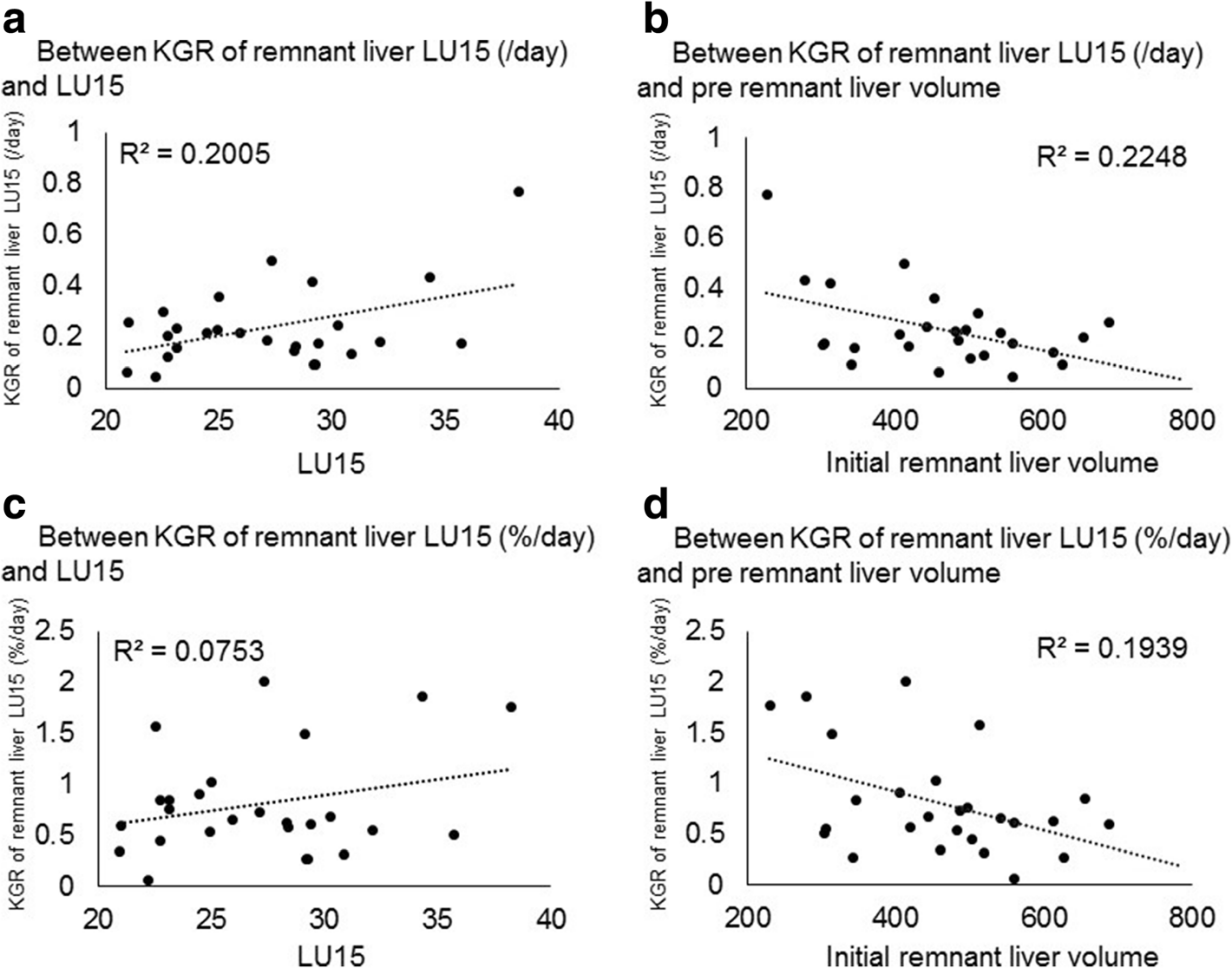
Correlation between kinetic liver growth rate (KGR) of remnant liver LU15 and LU15 or initial remnant liver volume. An inverse correlation between KGR of remnant liver LU15 and initial remnant liver volume was suggested. KGR of remnant liver LU15 decreased with increasing of the initial remnant liver volume. A positive correlation between KGR and LU15 was observed. Same trend was observed for KGR of remnant liver LU15 and initial remnant liver volume or LU15 as the correlation between KGR of liver volume and initial remnant liver volume or LU15 was seen

KGR (cc/day or %/day) of liver volume and LU15 or pre-operative remnant liver volume was plotted for all patients (Fig. 2). An inverse correlation between KGR of liver volume and initial remnant liver volume was suggested. KGR of liver volume decreased with increasing of the initial remnant liver volume. A positive correlation between KGR of liver volume and LU15 was observed. The relationship between KGR of remnant liver LU15 (/day or %/day) and initial remnant liver volume or LU15 was also plotted for all patients (Fig. 3). Same trend was observed for KGR of remnant liver LU15 and initial remnant liver volume or LU15 as the correlation between KGR of liver volume and initial remnant liver volume or LU15 was seen.

Out of all patients, two patients had liver failure. There was no association between KGR and liver failure in this study. There were no patient deaths within 90 days. Liver failure did not correlate with KGR or with KGR of remnant liver LU15.

## Discussion

Issues related to the size of the future remnant liver have become increasingly relevant in hepatic surgery as the envelope of resectability with larger and more complex hepatic resections continues to expand [14]. PVE is reliable and it has successfully increased the volume of the future remnant liver [15]. More recently the ALPPS procedure has also led to increases in the future remnant liver but the growth rate of ALPPS (34.8 cm^3^/day) compared with that of PVE (3 cm^3^/day) was 11 times greater [10]. In this research, KGR of ALPPS (19.0 cm^3^/day) was significantly greater than KGR of PVE (3.89 cm^3^/day), and same trend was observed in the remnant liver LU15 KGR. Importantly, we also showed that both remnant liver volume and function growth in the ALPPS was greater than in the PVE. The perspective presented by both variables of volume and function is novel, as most studies investigating liver hypertrophy report only volumetry of the remnant liver based on CT imaging [16].

We also sought to determine whether KGR was related to initial remnant liver volume and initial remnant liver total LU15. When KGR was plotted against the initial remnant liver volume, the correlation showed a clear linear trend of increasing KGR with a smaller remnant liver volume regardless of the procedure. These findings support the noteworthy observation that future remnant liver volume growth after ALPPS is marked. In fact, based on the volume of the future remnant liver, future remnant liver volume growth after ALPPS reflects a response that is expected after an extended hepatectomy. We have also demonstrated that both the volume and function of future remnant liver growth. Interestingly the correlation between KGR and initial total liver LU15 showed positive linear trend in all patients. In these findings, it was expected that the larger the initial total liver LU 15 and the smaller the initial remnant liver volume, the larger was KGR. Regarding the function, these results might be reasonable, because large LU 15 indicated that the remnant liver function was good.

Some authors have suggested that major liver resections should be avoided in patients with low KGR because these patients have a strongly increased risk of post-operative liver failure [15]. Interestingly others have shown that in selected patients with insufficient future remnant liver growth after PVE salvage the ALPPS approach has provided adequate growth [17]. In this study, the smaller the initial remnant liver volume, the greater was the KGR. No patient was seen with liver failure after PVE procedure. Small-for-size syndrome was only observed after liver resection surgery, especially after living donor liver transplantation. The reason why the situation of small-for-size syndrome was observed after the surgery, and not after PVE was unclear.

In conclusion, we have demonstrated a greater future remnant liver volume and KGR after the ALPPS procedure than after PVE. We have also demonstrated that KGR of patients undergoing the ALPPS procedure appears to be inversely related to the initial remnant liver volume and positively related to the initial total liver LU15. The important point of the present study is that liver hypertrophy is related to the initial remnant liver volume and total liver function.

## Conclusion

Future remnant liver volume and KGR was greater after the ALPPS procedure than after PVE. Liver hypertrophy is related to the expected remnant liver volume and total liver function. This study suggested that total liver function and initial remnant liver volume might be a new indication of hepatectomy after PVE and ALPPS in the case of insufficient remnant liver volume.
